# Intensive care unit prognostic factors in critically ill patients with advanced solid tumors: a 3-year retrospective study

**DOI:** 10.1186/s12885-016-2242-0

**Published:** 2016-03-05

**Authors:** Rui Xia, Donghao Wang

**Affiliations:** Key Laboratory of Cancer Prevention and Therapy, Intensive Care Unit, National Clinical Research Center of Cancer, Tianjin Medical University Cancer Institute and Hospital, Huanhu West Road, Ti-Yuan-Bei, Hexi District, Tianjin, 300060 China

**Keywords:** Advanced solid tumor, Intensive care unit, Mortality, Intensive care unit trial

## Abstract

**Background:**

The objective of this study was to identify risk factors predicting prognosis of critically ill medical patients with advanced solid tumors in the intensive care unit (ICU).

**Methods:**

We retrospectively analyzed all ICU unplanned medical admissions to the ICU of patients with advanced solid cancer in Tianjin Medical University Cancer Institute and Hospital between October 1, 2012 and March 1, 2015. Approval was obtained from the Ethical Commission of Tianjin Medical University Cancer Institute and Hospital to review and publish information from patients’ records.

**Results:**

One hundred and forty-one patients with full code status met the criteria for inclusion from among 813 ICU admissions. ICU mortality was 14.9 % and in-hospital mortality was 29.8 %. The major reasons for unplanned ICU admission were respiratory failure (38.3 %) and severe sepsis or septic shock (27.7 %). The ICU mortality in patients who required vasopressors, mechanical ventilation or renal replacement therapy for >24 h was 25, 25.9 and 40 %, respectively. The mean overall survival was 28.6 months. After adjusting for hypertension, type of solid cancer, intervention time, need for mechanical ventilation and Acute Physiology and Chronic Health Evaluation II score, only Sepsis-related Organ Failure Assessment (SOFA) score on day 7 of ICU treatment remained a significant predictor of ICU mortality (adjusted odds ratio 1.612, 95 % confidence interval 1.137–2.285, *P* = 0.007).

**Conclusions:**

We suggest broadening the criteria for ICU admission. The patients should be allowed an ICU trial consisting of unlimited ICU support, including invasive hemodynamic monitoring, mechanical ventilation and renal replacement therapy. An interdisciplinary meeting, including an ethics consultation, should be held to make end-of-life decisions if the SOFA score on day 7 shows clinical deterioration with no available therapeutic options.

## Background

Life expectancy is rising globally and the incidence of all-types of cancer is predicted to increase from 12.7 million new cases in 2008 to 22.2 million by 2030 [1]. An increasing number of older patients will live with tumors and acquire life-threatening complications from radical surgery, high-dose chemotherapy, adverse drug events [2], increased susceptibility to infection [3, 4] or cancer itself (such as tumor lysis syndrome, and hypercalcemia of malignancy) [5, 6]. As a consequence, there is an increase in critically ill patients with various types of malignancy at any stage requiring intensive care.

Cancer treatment near the end-of-life has become more aggressive and intensive care unit (ICU) mortality of cancer patients has improved in recent years [7–13]. However, patients with hematological or advanced-stage solid malignancies are still frequently denied admission to ICUs according to current policies, even if some of them may survive. Selection of patients inevitably leads to undertreatment and unnecessary deaths [11].

There have been few studies about unplanned ICU admission of critically ill patients with advanced solid tumors in China; therefore, we conducted this study to establish independent risk factors for prognosis in this patient subgroup. Three classic scoring systems on different lengths of stay in the ICU were compared for predicting prognosis. It will be helpful to identify patients who are most likely to benefit from critical care and decide the best time to terminate the ICU trial and discuss a change in code status.

## Methods

### Study design and setting

This was a retrospective single-center observational study conducted in the nine-bed general ICU managed by full-time faculty members of Critical Care of Tianjin Medical University Cancer Institute and Hospital, a 2400-bed hospital in Tianjin, China. All critically ill cancer patients admitted to the oncology general ICU were evaluated between October 1, 2012 and March 1, 2015. Patients who met all the following criteria were included: adult patients aged ≥18 years; medical patients with a definite diagnosis of solid cancer according to pathological results obtained by surgical or microinvasive biopsy; tumor metastasis assessed by radiography or exfoliative cytology; life expectancy evaluated by an oncologist as >3 months; >3 days in the ICU; and nonpregnant women. Medical oncologists conducted daily rounds on cancer patients in the ICU at the time of the study. Lymphoma was not included as a solid tumor in our study.

Epidemiological, clinical, and laboratory data collected from patients’ medical records and reports included: sex; age; time of ICU admission; chronic health status (history of chronic heart failure, diabetes mellitus, hypertension, chronic renal failure or chronic bronchitis); type of solid cancer; metastatic sites; history of anti-tumor therapy (such as chemotherapy, radiotherapy and biological therapy); Karnofsky Performance Status (KPS) at the time of admission to hospital and ICU; cause of ICU admission; time from physiological derangement to ICU intervention; Acute Physiology and Chronic Health Evaluation (APACHE) II and Sequential Organ Failure Assessment (SOFA) scores calculated from the worst values of physiological variables in the last 24 h on days 1, 3, and 7 of the ICU stay; presence and severity of sepsis upon ICU admission; site of infection and pathogens; therapeutic interventions during the ICU stay (use of vasopressors, mechanical ventilation or renal replacement therapy for >24 h); therapy after leaving ICU; length of ICU stay; ICU and in-hospital mortality; cause of death; and overall survival (OS). Code status on admission and day 3 and 7 of ICU stay was also included. Permission was obtained from the Ethical Commission of Tianjin Medical University Cancer Institute and Hospital to review and publish information from patients’ records. We had all necessary written consent from any patients involved in the study.

Patients with neutropenia (neutrophil count <500/mm^3^) were excluded from the study because of the absence of a laminar flow ward. Only insulin-treated patients were considered to have diabetes mellitus. Chronic renal failure was considered in patients requiring hemodialysis or peritoneal dialysis at the time of admission to the hospital. Chronic heart failure was defined as New York Heart Association grades III and IV [14]. Chronic bronchitis was defined as the presence of a productive cough or expectoration for >90 days a year (although on separate days) and for >2 (consecutive) years, provided that a specific disorder responsible for these symptoms was not present. Sepsis was defined as the presence of infection together with systemic manifestations of infection. Severe sepsis was defined as sepsis plus sepsis-induced organ dysfunction or tissue hypoperfusion. Septic shock was defined as persistent sepsis-induced hypotension despite adequate fluid resuscitation [15].

### Statistical analysis

Statistical analyses were performed using SPSS version 19.0 (SPSS Inc., Chicago, IL, USA). Numerical variables were described by using frequency statistics. Continuous variables were reported as median with interquartile range (IQR) according to the normality of distribution verified by Kolmogorov–Smirnov test. We examined between-group associations of demographic and clinical variables using the *χ*^2^ test for categorical variables, independent t test or t′ test for randomly distributed continuous variables, and the Mann–Whitney *U* test for non-normally distributed continuous variables. A logistic regression model was used to analyze the independent risk factors for prognosis in the ICU. Odds ratio (OR) and 95 % confidence interval (CI) were calculated using the Cox proportional hazards model to examine the effect of multiple factors on OS. All tests were two-sided, and *P* ≤ 0.05 was considered statistically significant. Variables yielding *P* ≤ 0.2 by univariate analysis and those considered clinically relevant were entered in the multivariate analysis to estimate the independent association of each covariate with the dependent variable.

## Results

### Characteristics of the study population

One hundred and forty-one patients met the inclusion criteria from among 813 ICU admissions during the study period. Their baseline characteristics are listed in Table 1. The main types of cancer were stomach cancer (23.4 %),pancreas cancer (12.8 %) and lung cancer (10.6 %). Adenocarcinoma was the most common pathological type (72 cases, 51.1 %). The top four metastatic sites were lung (21 cases, 14.9 %), bone (21 cases, 14.9 %), liver (18 cases, 12.8 %) and brain (12 cases, 8.5 %). The major reasons for unplanned ICU admission were respiratory failure (38.3 %) and severe sepsis or septic shock (27.7 %). Forty-five patients (31.9 %) were diagnosed with septic shock during ICU treatment. ICU mortality was 26.7 % (12 patients) and in-hospital mortality was 33.3 % (15 patients). Sixty patients (42.6 %) were diagnosed with severe sepsis in the ICU. ICU mortality was 10 % (six patients) and in-hospital mortality was 35 % (21 patients). The main infections were pneumonia(66 cases, 62.9 %), abdominal infection (27 cases, 25.7 %) and urinary tract infection (nine cases, 8.6 %). The most common pathogens cultured from blood, sputum, bronchoalveolar lavage fluid or normally sterile sites were *Klebsiella pneumoniae* (21 cases, 20 %), *Pseudomonas aeruginosa* (15 cases, 14.3 %) and *Candida tropicalis* (12 cases, 11.4 %). Sixty patients (42.6 %) required vasopressors for >24 h, 81 (57.4 %) mechanical ventilation, and 15 (10.6 %) renal replacement therapy.

**Table 1:** Characteristics and outcomes of medical patients with advanced solid cancer in the ICU

Variables	N (%) or median (25^th^-75^th^ percentile)
Age	63 (54–74)
Sex (male)	87 (61.7 %)
Chronic health status	
Diabetes mellitus	57 (40.4 %)
Chronic heart failure	48 (34 %)
Hypertension	48 (34 %)
Chronic renal failure	0 (0)
Chronic bronchitis	6 (4.3 %)
Types of solid cancer	
Stomach cancer	33 (23.4 %)
Pancreas cancer	19 (13.5 %)
Lung cancer	15 (10.6 %)
Rectal cancer	10 (7.1 %)
Colon cancer	10 (7.1)
Esophageal cancer	6 (4.3 %)
Breast cancer	6 (4.3 %)
others	42 (29.8 %)
History of antitumor therapy	
Surgery	123 (87.2 %)
Chemotherapy	72 (51.1 %)
radiotherapy	18 (12.8 %)
biological therapy	6 (4.3 %)
KPS	
Admit to hospital	80 (50–90)
Admit to ICU	10 (10–10)
Intervention time (hours)	3 (2–12)
Major reasons for ICU	
Respiratory failure	54 (38.3 %)
Severe sepsis or septic shock	39 (27.7 %)
Acute renal failure	12 (8.5 %)
Acute heart failure	18 (12.8 %)
ICU therapeutic interventions	
vasopressors	60 (42.6 %)
mechanical ventilation	81 (57.4 %)
renal replacement therapy	15 (10.6 %)
APCHE II score	
Day 1	21 (18–28)
Day 3	14 (11–20)
Day 7	12 (10–15)
SOFA score	
Day 1	9 (5–13)
Day 3	5 (3–9)
Day 7	4 (2–7)
Outcomes	
Length of ICU stay (days)	6 (3–10)
ICU mortality	21 (14.9 %)
In-hospital mortality	42 (29.8 %)
Overall survival (month)	17 (4–27)

Median time to intervention was 3 (IQR2–12) h.

### Outcomes

The ICU mortality was 14.9 % (21 of 141 patients) and the in-hospital mortality was 29.8 % (42 of 141 patients). The ICU mortality of all 813 patients and other surgical patients during the study period was 4.3 and 2.1 %, respectively. The ICU mortality in patients who required vasopressors, mechanical ventilation or renal replacement therapy for >24 h was 25, 25.9 and 40 %, respectively. The in-hospital mortality in patients who required vasopressors, mechanical ventilation or renal replacement therapy for >24 h was 35, 44.4 and 40 %, respectively. The mean OS was 28.6 months. The median length of the stay in the ICU was 6 (IQR3–10) days. Fifteen patients (10.6 %) received chemotherapy, 12 patients (8.5 %) received radiotherapy, and three (2.1 %) received palliative surgery after discharge from the ICU.

All of the patients lacked decision-making capacity and had surrogates. The code status of all patients upon ICU admission was full code. Nine patients changed their goals on day 3 in the ICU because of worsening medical conditions. Three surrogates (2.1 %) changed to palliative care. Six surrogates (4.2 %) changed to supportive care. They decided to withdraw treatment and implemented do-not-resuscitate, and three died in the ICU and the other three in a general ward. Twelve surrogates (8.4 %) changed goals on day 6–7 to palliative care after the ICU trial. Three of them died in the ICU, and six in a general ward, and three were discharged from hospital. Three surrogates changed to supportive care on day 6 in the ICU. The difference between palliative care and supportive care lied in the fact that the latter was mainly provided by ICU team by means of life-sustaining treatment regardless of prognosis, while the former relied more on the nutrition support and family care which could be undertaken in the general ward or at home. Twenty-one patients (14.9 %) died without changing code status: 15 from tumor rupture bleeding and six from cardiogenic shock (Table 2).

**Table 2:** Patient care decisions in the ICU

ICU days	Palliative care	Supportive care	Intensive care
Day 3	3 (2.1 %)	6 (4.2 %)	132 (93.6 %)
Day 6–7	12 (8.4 %)	3 (2.1 %)	117 (83.0 %)

### Univariate analysis

Univariate comparisons of the clinical characteristics and outcomes of survivors and non-survivors in the ICU are presented in Table 3. Age, APACHEII score on days 1 and 3, and SOFA score on days 1, 3 and 7 were normally distributed in survivors and non-survivors, and verified by the Kolmogorov–Smirnov test. Mean OS was 30.7 months in survivors and 16.7 months in non-survivors. Median time to ICU intervention was significantly shorter in survivors than in non-survivors (3 vs 24 h). APACHEIIand SOFA scores on days 1, 3, and 7 of ICU treatment were significantly higher in non-survivors. Other factors associated with higher ICU mortality were non-stomach cancer, lung cancer, history of hypertension, and need for mechanical ventilation.

**Table 3:** Characteristics and outcomes of survivors and non-survivors medical patients with advanced solid cancer in the ICU

Variables	Survivor (*n* = 120)	Non-survivor (*n* = 21)	*P* value
Age	62 (54–73)	64 (55–74)	0.448
Sex (male)	72 (60 %)	15 (71.4 %)	0.692
Chronic health status			
Diabetes mellitus	48 (40 %)	9 (42.9 %)	1.000
Chronic heart failure	45 (37.5 %)	3 (14.3 %)	0.396
Hypertension	27 (22.5 %)	18 (85.7 %)	**0.003**
Chronic renal failure	0 (0)	0 (0)	-
Chronic bronchitis	6 (5 %)	0 (0)	1.000
Types of solid cancer			
Stomach cancer	33 (27.5 %)	0 (0)	**0.004**
Pancreas cancer	19 (15.8 %)	0 (0)	0.075
Lung cancer	9 (7.5 %)	6 (28.6 %)	**0.011**
Rectal cancer	10 (8.3 %)	0 (0)	0.359
Colon cancer	8 (6.7 %)	2 (9.5 %)	0.644
Esophageal cancer	4 (3.3 %)	2 (9.5 %)	0.219
Breast cancer	6 (5 %)	0 (0)	0.592
others	32 (26.7 %)	10 (47.6 %)	0.066
History of antitumor therapy			
Surgery	108 (90 %)	15 (71.4 %)	0.214
Chemotherapy	60 (50 %)	12 (57.1 %)	1.000
radiotherapy	15 (12.5 %)	3 (14.3 %)	1.000
biological therapy	6 (5 %)	0 (0)	1.000
KPS			
Admit to hospital	80 (50–90)	40 (20–90)	0.198
Admit to ICU	10 (10–10)	10 (10–10)	0.310
Intervention time (hours)	3 (1.3–7)	24 (3–52)	**0.028**
Major reasons for ICU			
Respiratory failure	42 (35 %)	12 (57.1 %)	0.403
Severe sepsis or septic shock	39 (32.5 %)	0 (0)	0.166
Acute renal failure	6 (5 %)	6 (28.6 %)	0.100
Acute heart failure	18 (15 %)	0 (0)	0.571
ICU therapeutic interventions			
vasopressors	45 (37.5 %)	15 (71.4 %)	0.119
mechanical ventilation	60 (50 %)	21 (100 %)	**0.015**
renal replacement therapy	9 (7.5 %)	6 (28.6 %)	0.154
APCHE II score			
Day 1	20 (17–25)	28 (26–35)	**0.001**
Day 3	13 (10.25–16.75)	29 (22–40)	**0.000**
Day 7	11.5 (10–13.75)	35 (23–41)	**0.000**
SOFA score			
Day 1	7.5 (5–11.75)	14 (12–19)	**0.002**
Day 3	4.5 (2–7)	17 (10–18)	**0.000**
Day 7	3 (1–5)	17 (11–23)	**0.000**
Outcome			
Length of ICU stay (days)	7 (5–10)	3 (3–8)	0.220
Overall survival (month)	18.5 (4.25–31.5)	6 (3–24)	0.324

The bold symbol: *P *<0.05

### Multivariate analysis

After adjusting for hypertension, intervention time, need for mechanical ventilation, APACHEII score, and other variables yielding *P* ≤ 0.2, only SOFA score on day 7 of ICU treatment remained a significant predictor of ICU mortality. (adjusted OR 1.612, 95 % CI 1.137–2.285, *P* = 0.007).

APACHEII score on day 1 (adjusted OR 0.771, 95 % CI 0.603–0.987, *P =* 0.039) was the independent risk factor of OS assessed by Cox regression analysis.

## Discussion

Recently, Gruber and co-workers reported a 12-month mortality rate of 48.3 % for long-stay ICU patients with cancer, which means that more than half of long-stay critically ill cancer patients survive ≥1 year [10]. Many studies have documented improved survival of critically ill patients with cancer. Two main hypotheses have been proposed to account for the decreased mortality rate. First, the development of more potent and targeted anti-tumor therapies, advances in the standard strategies for determining indications and supportive care, as well as progress in the prevention of organ dysfunction. Cancer patients benefit from reduced cancer-related complications or timely intervention. Second, with a deeper understanding of the pathophysiological mechanisms in organ dysfunction, intensive care has improved survival of critical illness by constantly renewing strategies for survival of sepsis, hemodynamic monitoring, mechanical ventilation, nutrition support, sedation, and analgesia [5, 12, 16].

The in-hospital mortality of patients with solid cancer in our study was similar to that reported from European ICUs [4]. The crude ICU mortality was 14.9 %. The ICU mortality of patients diagnosed with septic shock was 26.7 and 10 % in those diagnosed with severe sepsis. The ICU mortality of patients who required vasopressors, mechanical ventilation or renal replacement therapy for >24 h was 25, 25.9 and 40 %, respectively. When patients were admitted to the ICU, their APACHEII or SOFA scores were comparable to those from most previous studies. However, the reason why the ICU mortality rate observed in our study mentioned above was lower than previously 30–70 % was multifactorial [4, 17–21], including different underlying diseases, types of cancer, and ICU admission or discharge criteria. As patients with early-stage solid tumors after elective surgery were the main group in our ICU, the higher mortality of cancer patients admitted for medical reasons was also observed (14.9 % vs 2.1 %) [21].

As intensive care specialists, we should realize that the endpoint of therapy in patients with advanced-stage cancer differs from that in patients without cancer. We should not be concerned only with survival rate but also with long-term survival and quality of life [6]. During our study, in-hospital survival reached nearly 70 % after a median 6 days in the ICU. APACHEII score on day 1 predicted poor OS, but the mean OS had already reached 28.6 months. Thirty patients (21.3 %) had the opportunity to receive anti-cancer treatment after ICU treatment. Active treatment in the ICU could be more important than many anti-cancer therapies if offers the possibility of prolonging survival with good quality of life for >3 months. In fact, we reached this outcome after a median 6 days of ICU treatment.

Patients with advanced-stage cancer are frequently denied admission to ICUs that are normally run by non-oncologists according to current policy. Several studies have failed to show that diagnosis or stage of cancer is an independent predictor of ICU mortality [4, 9, 10, 16, 18, 19, 22–24], which was confirmed by our logistic regression model. In other words, triage decisions solely based on the type of cancer are thus not justified. Intensivists sometimes need to make quick decisions based on little or inconclusive information. Sometimes, we may find a high hospital survival rate in a small number of patients for whom an agreement to limit care was not achieved [25]. Thiéry and co-workers showed 26 % survival on day 30 in patients who were considered too ill to benefit from ICU admission. Among the patients who were denied ICU admission because they were felt to be too well to benefit from admission, one quarter were subsequently admitted, and mortality was high (61.5 %) in this subgroup [26]. Rapid selection depending on unreliable triage criteria will inevitably lead to undertreatment and unnecessary death in a minority of patients [11]. The balance between reasonable hope of benefit and excessive burdens on the family or community urgently requires an effective oncology critical scoring system and risk factors analysis to broaden ICU admission criteria for patients with cancer. APACHEIIand SOFA are the most commonly used scoring systems in the ICU, while Eastern Cooperative Oncology Group performance status (ECOG-PS) or KPS is often used in oncology departments to evaluate indications for anti-tumor therapy. SOFA score on day 3–6 in the ICU [4, 17–19, 23, 27] and ECOG-PS [17, 22, 28, 29] are frequently mentioned as significant risk factors for prognosis. In our study, SOFA score on day 7 of ICU treatment was assessed to be the only significant predictor of ICU mortality, which means that poor performance on admission plays a limited role in the ICU decision-making process. In fact, the severity of physiological derangement in the subsequent 6 days from ICU admission has the biggest impact on ICU survival. We should receive more often than refuse selected patients with cancer for ICU admission [16]. An ICU trial should be offered in particular during the first week of ICU stay [19].

The ICU trial is considered as an alternative to ICU refusal for patients with cancer. It consists of unlimited ICU support, including ambulatory chemotherapy, along with mechanical ventilation and renal replacement therapy, for a limited time period [5, 11, 28]. After the defined 6 days, an interdisciplinary meeting consisting of oncologists, intensivists, nurses, psychologists, and palliative care, pain, and ethics specialists should be held. The treatment goals should shift from curative or supportive therapies to end-of-life care if the reevaluation on day 7 shows clinical deterioration with no available therapeutic options [25]. This decision to limit treatment should be based on certainty of the benefits of the applied treatment and that it does no harm according to the 5th International Consensus Conference in Critical Care [30]. By strengthening the interdisciplinary collaboration to enhance advantages and minimize disadvantages, we could integrate hospice and palliative care with intensive care more effectively and efficiently. That will be the future of oncological ICUs [31–33].

Our study had several limitations. First, this was a retrospective study at a single cancer center. However, to the best knowledge, it is the first report about the prognosis and risk factors of critically ill patients with advanced solid tumor in the ICU in China. Second, the small size of the sample prevented us from investigating the characteristics of critical illness in patients with different types of solid cancer and the effect of ambulatory chemotherapy. Third, early identification and treatment of critically ill cancer patients on general wards showed no significance in our study, which was contrary to the results from many previous studies. This is probably because the medical emergency team, which facilitates early intervention in response to physiological instability, was not standard in our hospital [14, 16, 18, 23]. The intervention time in our records may have been shorter than the real intervention time. Fourth, ethical consultation is not yet ideal in our hospital. Twenty-one patients in full code status died from an emergency at the end of life because of the lack of ethical consultation at the time of ICU admission. Our results needs to be confirmed by a large prospective study.

## Conclusion

In summary, an increasing number of cancer patients require intensive care. The success of active ICU treatment may offer them the opportunity to prolong survival with good quality of life and receive effective anti-cancer therapy. According to traditional ICU admission criteria, critically ill patients with advanced solid tumors are often deprived of the opportunity for intensive care, even though >70 % of them would benefit if admitted. We suggest broadening the criteria for ICU admission. Patients should be allowed an ICU trial that consists of unlimited ICU support, including invasive hemodynamic monitoring, mechanical ventilation, and renal replacement therapy. An interdisciplinary meeting including ethics consultations should be held to make clinical decisions if the SOFA score on day 7 shows clinical deterioration with no available therapeutic options. The goal of the treatment may shift from curative or supportive therapy to end-of-life care.

## Abbreviations

APACHE: acute physiology and chronic health evaluation
CI: confidence interval
ICU: intensive care unit
IQR: interquartile range
KPS: karnofsky performance status scale
OR: odds ratio
OS: overall survival
SOFA: sequential organ failure assessment
